# Transactions of California State Dental Association

**Published:** 1873-12

**Authors:** 


*Transactions of the California State Dental Association.* First, Second, Third and Fourth Annual Sessions, 1870–71–72–73, held at San Francisco.

This is a very handsome record of 217 pages, for which we are indebted to Dr. H. L. Plomteaux, Secretary of the Association.

